# Resident Research in Emergency Medicine: An Introduction and Primer

**DOI:** 10.5811/westjem.2020.6.46520

**Published:** 2020-08-24

**Authors:** James H. Paxton, Anne M. Messman, Nicholas E. Harrison, Adrienne N. Malik, Raina J. Burke, Phillip D. Levy

**Affiliations:** * Wayne State University, Department of Emergency Medicine, Detroit, Michigan; † Kansas University Medical Center, Department of Emergency Medicine, Kansas City, Kansas; ‡ Wheeling Hospital, Department of Emergency Medicine, Wheeling, West Virginia

## Abstract

Training in research methodology represents an important aspect of emergency medicine (EM) resident education, but best methods for design, implementation, and dissemination of resident research remain elusive. Here we describe recommendations and best practices from the existing literature on EM resident research, including helpful tips on how to best implement a resident research program.

## INTRODUCTION

When René Laënnec, a French physician in 1816, failed to adequately percuss the thorax of a young woman with heart disease, he improvised. Laënnec wrote, “I rolled a quire of paper into a sort of cylinder and applied one end of it to the region of the heart and the other to my ear.”^1^ After numerous revisions, his invention was revealed to the medical community, and quickly caught on. Within a few years, primitive stethoscopes could be found in medical shops throughout Paris. Had Laënnec stopped with that rolled-up piece of paper, his one-time improvisation would have been lost to the annals of history. Fortunately, he chose to build upon his initial discovery and, crucially, to share his breakthrough with the world. Laënnec’s journey charts an enduring and fundamental trajectory of medical innovation: from observation, through inspiration, refinement and testing, to dissemination.

Development of a research project can be especially daunting to physicians already engaged in an emergency medicine (EM) residency training program. But execution of a research project during residency remains a worthwhile experience, allowing participants to meaningfully contribute to medical knowledge and develop an investigative spirit.^2^ Residents participating in research appear to attain greater job satisfaction,^3^ and can objectively frame everyday questions and methodically seek answers^3^ to problems including (among others) staffing issues, wait times, and communication barriers.^4^–^5^

The Accreditation Council for Graduate Medical Education Residency Review Committee for EM recognizes the importance of these efforts, mandating resident completion of a “scholarly project” prior to graduation. Their requirement cites the following as examples of qualifying activities: “…the preparation of a scholarly paper such as a collective review or case report, active participation in a research project or formulation and implementation of an original research project.”^6^ These activities should include problem identification, data collection, analysis, and conclusion.^7^ Performance and documentation of these projects are vital to the acceptance of a scholarly project, whether a case report, community project, development of medical software, or traditional research project.^7^ Recent reports from within the EM community have emphasized the importance of scholarly activity to EM resident education.^8^–^9^

Advancing the state of scientific knowledge is not a requirement for success in resident research, but it is a potential benefit of this exercise. It is the responsibility and privilege of those involved in residency administration to facilitate the training of EM resident researchers in the development and execution of research projects that support not only the professional careers of residents but also the advancement of our specialty.^8^–^9^

### What is Resident Research?

It has been suggested that “resident research” is, “research where a resident has a principal role in the implementation and completion of the project.”^10^ We suggest that the resident research experience be defined by the engagement of the resident learner in the research process, focusing upon the educational value of the project rather than the resident’s official role or involvement in the design and execution of the project. Research studies are intended to create *new generalizable knowledge* that can be applied to other populations and settings.^11^ Consequently, we propose that “resident research” be defined as any systematic investigation designed to yield new information that actively engages the resident-learner and facilitates the acquisition of a greater understanding of the scientific method. This is in distinction to quality improvement projects, which seek to apply existing knowledge to improve healthcare outcomes within a local healthcare institution or setting.^12^

### Setting Realistic Expectations

One purpose of resident research is to expose residents to the methods by which research is conducted, creating “educated consumers” of the medical literature. However, residencies hoping to establish a resident research program de novo must recognize the additional workload that resident research projects impose upon faculty. Mentors should be primarily responsible for guiding and supervising resident research, but should be adequately vetted to ensure that the research experience yields a positive result for all involved. Research directors should provide guidance relating to funding opportunities, deadlines for abstract submission to key research conferences, important institutional and federal regulations, and departmental resources.^13^ Departmental leadership should create an environment in which research is actively promoted, providing appropriate funding and protected time for mentors and other research faculty.^13^

### Getting Started

Clinical experiences, journal club articles, or experiences with different teaching modalities may generate an appropriate resident research topic including relevant clinical or educational questions.^2^, ^14^–^15^ Additional ideas may come from the resident’s personal interests or experiences.

### Learning Research Methodology

Most programs will offer training through didactic presentations, journal clubs or evidence-based literature discussions. However, a focused educational effort specifically targeting research methodologies has been shown to correlate with improved resident skills, knowledge, and research productivity.^16^ Nearly one in four EM training programs offers a fixed rotation in research.^5^,^17^ A more feasible format for the busy trainee might be the Advanced Research Methodology Evaluation and Design video series available from the Society of Academic Emergency Medicine (SAEM), including “how-to” webinars and podcasts produced by senior researchers.^18^

### The Research Question

A general research question must be formulated, which will generate a testable hypothesis.^3^,^19^ All possible outcomes should be considered, and at least one of them must be worthwhile.^20^ The FINER criteria may be used to assess the relative merits of the proposal:^14^–^15^,^21^

**F**easible
  Can the project be completed within the time allotted using the given resources? Can the proposed investigation enroll enough patients to demonstrate a difference in the proposed outcome measures?
**I**nteresting
  Is the topic engaging enough to be worth the effort?
**N**ovel
  Is the proposed investigation different enough from what has been done before to add knowledge on the subject?
**E**thical
  Does the proposed investigation respect the morals of the community, the patient, and the profession?
**R**elevant
  Are the results likely to be applicable to many patients? Will the results be useful and contribute to the greater good?

### Formulating a Hypothesis

A suitably refined and meaningful research question will help in generating a hypothesis, providing a clear delineation of what the investigation will attempt to prove. Investigation of a well-designed hypothesis will be interesting even if a negative result is found.

### The Mentor

A mentor experienced in the resident’s area of research interest can be an invaluable resource by offering hints at project scope, helping with setbacks, and tailoring the learning experience to the resident’s needs.^22^ Most often, the mentor is an established researcher within the department but could include a specialist in another field, or even a non-physician investigator.^5^, ^23^–^24^ Goals and expectations should be discussed early on, to avoid frustration for both parties.^5^ Terregino has shown that, in general, EM residents are relatively unfamiliar with what resources are available to them, which can lead to significant amounts of time wasted.^25^ Most hospitals provide research support that is invisible to the outside observer, including project coordinators, departmental research directors, and biostatisticians.^25^ The mentor should be aware of all available institutional resources.

### The Literature Search

A valid research project must be informed by past work. Most literature reviews will begin with a search of PubMed.gov, the database of the National Library of Medicine, or OVID.org, which includes textbooks as well as journals.^26^ Search terms used must be carefully selected, and the proper Boolean operators assigned. One study has shown physicians to be especially inept at crafting effective search strings.^27^ Any doubts about the literature search process or its results should be referred to a librarian.

Each paper identified from the literature review should be thoroughly read. Investigators should avoid citing abstracts alone, as they are often incomplete in their data presentation. This process is labor-intensive but necessary to form a strong foundation for the research project. All references cited within each article should be assessed for relevance. The selected literature should be reviewed to better understand the subject matter and to develop context for the proposed work. If adequate data from existing sources are uncovered, one may consider a retrospective evaluation of prior results including a meta-analysis.^2^, ^28^–^29^

### Research Design

The novice researcher should look to the existing medical literature for guidance in how to properly design a new study. Selection of the proper research methodology will depend upon multiple factors, including the research question, hypothesis, and predetermined outcome measures. A timeline should be implemented to ensure that all tasks are achievable within the allotted time. Resident physicians should develop a team approach, incorporating input from the faulty mentor as well as a staff epidemiologist or biostatistician. The required sample size will depend upon a variety of factors, including the acceptable level of significance, power of the study, expected effect size, underlying event rate in the population, and standard deviation in the population.^30^–^31^ Efforts should be made to collect an inclusive and truly random sampling, to avoid convenience selection bias.^32^ Early consultation with the biostatistician will also inform the researcher’s decisions on the most appropriate methods for the statistical analysis of data derived from the study. For further information about study design specifics, the reader is referred to several existing publications.^2^, ^4^, ^13^, ^33^–^34^

### The Institutional Review Board

Any research project that involves human participants or their data requires submission to the local institutional review board (IRB). Research protocols submitted to the IRB can fall into one of three categories: full submission; expedited; or exempt. Research involving greater than minimal risk to human subjects will require a thorough review by the IRB and development of an informed consent document. Prospective projects involving only minimal risk may be approved via the expedited process, where a single reviewer may approve the work in lieu of the convened board. Studies that include only retrospective data from the electronic health record may be exempt from IRB review, but this determination should be made by the IRB, rather than by the investigator. Investigators should confer with their local IRB to confirm what level of IRB review is required before beginning data collection.

### Conducting the Study

After the research protocol has been IRB-approved or exempted, data collection can commence. Prior development of a data collection tool will greatly enhance the efficiency of this process, facilitating both IRB approval and the subsequent data analysis. Subject enrollment can also be improved with use of a trained research assistant. This problem may be circumvented through creation of an “academic associate program,” which integrates EM research with undergraduate education.^35^

### Research Funding

Resident research projects usually require little external funding. On occasion, additional costs may be incurred to help pay for statistical analysis, or the purchase of required equipment.^36^ Internal sources, as well as the Emergency Medicine Foundation 37 and the SAEM Foundation 38 represent potential sources for funding.

### Presentation and Publication

Once the data have been collected and analyzed, the researcher should consider how the results will be disseminated. The annual meeting of SAEM, the Research Forum at the American College of Emergency Physicians’ annual scientific assembly, and the Annual Assembly of the Council of Residency Directors in Emergency Medicine (CORD) represent the premier locales for presentation of EM research.^39^

Ideally, the resident research experience should lead to a manuscript, although the lack of immediate publication must not be interpreted as failure. Only 40% of EM abstracts go on to become full article publications.^15^,^40^ Most manuscripts are published 1–2 years after initial presentation.^17^ Appropriate journal selection for submission enhances the likelihood of success, as does a thorough understanding of manuscript preparation techniques and review criteria.^41^–^43^

## CONCLUSION

While any research resultant from a resident’s scholarly project is unlikely to have the impact of Laënnec’s stethoscope, EM residents may still gain much from engaging in clinical research. For some, it will light an investigative fire that will burn for an entire career. At the least, resident research projects can provide an opportunity to explore issues central to the practice of EM, helping the resident to become a more well-rounded physician.

## References

[1] Simmons J (2002). Doctors and Discoveries.

[2] Wyatt J, Guly H (2002). Identifying the research question and planning the project. Emerg Med J.

[3] Durbin CG (2004). How to come up with a good research question: framing the hypothesis. Respir Care.

[4] Goodacre S (2003). Research methods: beyond the clinical trial. Ann Emerg Med.

[5] Zink B (2002). Resident Research: The basics and beyond. Newsletter of the Society of Academic Emergency Medicine.

[6] Accreditation Council for Graduate Medical Education (2020). ACGME Program Requirements for Graduate Medical Education in Emergency Medicine.

[7] Summers RL, Fish S, Blanda M (1999). Assessment of the “scholarly project” requirement for emergency medicine residents: report of the SAEM Research Directors’ workshop. Acad Emerg Med.

[8] Kane BG, Totten VY, Kraus CK (2019). Creating consensus: Revisiting the emergency medicine resident scholarly activity requirement. West J Emerg Med.

[9] Langabeer J, Gottlieb M, Kraus CK (2018). Scholarship in emergency medicine: A primer for junior academics: Part II: Promoting your career and achieving your goals. West J Emerg Med.

[10] Levittt MA (1997). Can emergency medicine residents perform quality research?. Acad Emerg Med.

[11] Office for Human Research Protections, US Department of Health and Human Services Code of Federal Regulations. 45 CFR 46.

[12] Institute of Medicine (US) Committee on Quality of Healthcare in America (2001). Crossing the quality chasm: A new health system for the 21st century.

[13] Biros MH, Barsan WG, Lewis RJ (1998). Supporting emergency medicine research: Developing the infrastructure. Ann Emerg Med.

[14] Pollack C, Wadbrook P (1999). Successful resident research in emergency medicine: the keys to relevance and quality. Ann Emerg Med.

[15] Good AM, Driscoll P (2002). Clinical research in emergency medicine: putting it together. Emerg Med J.

[16] Rydman RJ, Zalenski RJ, Fagan JK (1994). An evaluation of research training in a large residency program. Acad Emerg Med.

[17] Chacko S, Banet G, Narsahimhalu K (2003). Predictors of residency program scholarly productivity. Acad Emerg Med.

[18] Society for Academic Emergency Medicine (SAEM) (2020). Advanced research methodology, evaluation and design (ARMED) website.

[19] Kwiatkowski T, Silverman R (1998). Research fundamentals: II. Choosing and defining a research question. Acad Emerg Med.

[20] Kahn CR (1994). Picking a research problem. The critical decision. N Engl J Med.

[21] Hulley S, Cummings ST (1988). Designing Clinical Research.

[22] Panchal AR, Denninghoff KR, Munger B (2014). Scholar quest: A residency research program aligned with faculty goals. West J Emerg Med.

[23] Biros MH (2000). Reforming a solitary passion. Acad Emerg Med.

[24] Zink B (2000). Emergency Medicine Research - no more excuses. Newsletter of the Society of Academic Emergency Medicine.

[25] Terregino CA, Levitt MA, Lopez BL (1999). A national profile of resident research experience. Acad Emerg Med.

[26] Rau JL (2004). Searching the literature and selecting the right references. Respir Care.

[27] Bronander KA, Goodman PH, Inman TF (2004). Boolean search experience and abilities of medical students and practicing physicians. Teach Learn Med.

[28] Davey Smith G, Egger M (1998). Meta-analysis. Unresolved issues and future developments. Br Med J.

[29] Egger M, Schneider M, Davey Smith G (1998). Spurious precision? Meta-analysis of observational studies. Br Med J.

[30] Kirby A, Gebski V, Keech AC (2002). Determining the sample size in a clinical trial. Med J Aust.

[31] Kadam P, Bhalerao S (2010). Sample size calculation. Int J Ayurveda Res.

[32] Simundic A-M (2013). Bias in research. Biochem Med (Zagreb).

[33] Silverman R, Kwiatkowski T (1998). Research fundamentals: III. elements of a research protocol for clinical trials. Acad Emerg Med.

[34] Jones JB (2000). Research fundamentals: statistical considerations in research design: a simple person’s approach. Acad Emerg Med.

[35] Hollander JE, Singer AJ (2002). An innovative strategy for conducting clinical research: the academic associate program. Acad Emerg Med.

[36] Carden DL, Dronen SC, Gehrig G (1998). Funding strategies for emergency medicine research. Ann Emerg Med.

[37] Emergency Medicine Foundation (2020). Apply for a grant.

[38] Society for Academic Emergency Medicine (SAEM) Foundation (2020). What we fund.

[39] Singer AJ, Homan CS, Brody M (1999). Evolution of abstracts presented at the annual scientific meetings of academic emergency medicine. Am J Emerg Med.

[40] Davenport M, Grudzen C, Rao R (2004). Publication rates in emergency medicine: A comparison among specialties. Acad Emerg Med.

[41] Bordage G, Caelleigh AS, Steinecke A (2001). Review criteria for research manuscripts. Acad Med.

[42] DeBehnke DJ, Kline JA, Shih RD (2001). Research fundamentals: choosing an appropriate journal, manuscript preparation, and interactions with editors. Acad Emerg Med.

[43] Gottlieb M, Lotfipour S, Murphy L (2018). Scholarship in emergency medicine: A primer for junior academics. Part I: Writing and publishing. West J Emerg Med.

